# Co-existence of a giant splenic hemangioma and multiple hepatic hemangiomas and the potential association with the use of oral contraceptives: a case report

**DOI:** 10.1186/1752-1947-2-147

**Published:** 2008-05-07

**Authors:** George Chatzoulis, Andreas Kaltsas, Stauros Daliakopoulos, Osama Sallam, Kaltsa Maria, Kostas Chatzoulis, Ioannis Pachiadakis

**Affiliations:** 1 Department of Surgery, 424 Military Hospital, Grigoriou Labraki 3, 54636 Thessaloniki, Greece; 2 Department of Pathology St Loukas Hospital Thessaloniki, Greece; 3 Department of Gastroenterology, 424 Military Hospital Thessaloniki, Greece; 4 Department of Linguistics, University of Cambridge, UK

## Abstract

**Introduction:**

Hepatic and splenic hemangiomas are common benign tumors that mainly affect female patients. Giant splenic hemangiomas are extremely rare, especially when correlated with multiple hepatic hemangiomas. Pathogenetic mechanisms between hemangiomas and oral contraceptives, as well as therapeutic approaches, are analyzed in this case report, in particular for the management of synchronous splenic and hepatic hemangiomas.

**Case presentation:**

We report here a 42-year-old woman with a giant splenic hemangioma, multiple hepatic hemangiomas and a history of oral estrogen intake for many years. At first it was difficult to determine the organ from which the giant hemangioma originated. Angiography proved extremely helpful in tracing its origin in the spleen. Hematomas in the giant hemangioma posed a significant threat of rupture and catastrophic hemorrhage. We left the small hepatic hemangiomas in place, and removed the spleen along with the giant splenic hemangioma.

**Conclusion:**

Diagnostic pitfalls in the determination of the origin of this giant hemangioma, attribution of its origin to the spleen angiographically, the unusual co-existence of the giant splenic hemangioma with multiple hepatic ones, and the potential threat of rupture of the giant hemangioma are some of the highlights of this case report. Estrogen administration represents a pathogenic factor that has been associated with hemangiomas in solid organs of the abdominal cavity. The therapeutic dilemma between resection and embolization of giant hemangiomas is another point of discussion in this case report. Splenectomy for the giant splenic hemangioma eliminates the risk of rupture and malignant degeneration, whereas observation for the small hepatic ones (<4 cm) was the preferable therapeutic strategy in our patient.

## Introduction

Splenic hemangioma is a vascular malformation that represents the most common benign primary neoplasm of the spleen. Its prevalence at autopsy ranges from 0.03% to 14% and it is the hemangioma most commonly seen in adults in the mid-30- to mid-50-year-old range [1].

Most hemangiomas are small asymptomatic lesions that are found incidentally. Previous studies have noted liver and spleen hemangioma augmentation during estrogen administration for endometriosis treatment, revealing a potential association [2]. Additionally the co-existence of benign hepatic and giant splenic hemangiomas is extremely rare. To our knowledge this is the first bibliographic case report of this combination.

## Case presentation

A 42-year-old woman presented with a left upper abdominal distention, early satiety and discomfort. There was no family history of hematological or splenic disorders. There was no evidence of thrombocytopenia, hemorrhagic episodes, hepatomegaly or lymphadenopathy. The patient had received estrogen therapy as treatment for endometriosis for 10 years, but she discontinued this medication 12 years prior to her presentation to our unit. The patient had a palpable mass in her left upper abdomen. The mass could be palpated approximately 9 cm below the left costal margin. Her full blood count was in the normal range.

Initially a spiral computed tomography (CT) scan with IV contrast showed multiple hypodense lesions with centripetal filling in delayed venous phase compatible with hemangiomas. Two large lesions were located in the right hepatic lobe and multiple smaller ones in the caudate and left lobe. All these hepatic lesions were consistent with the features of hemangiomas.

**Figure 1:**
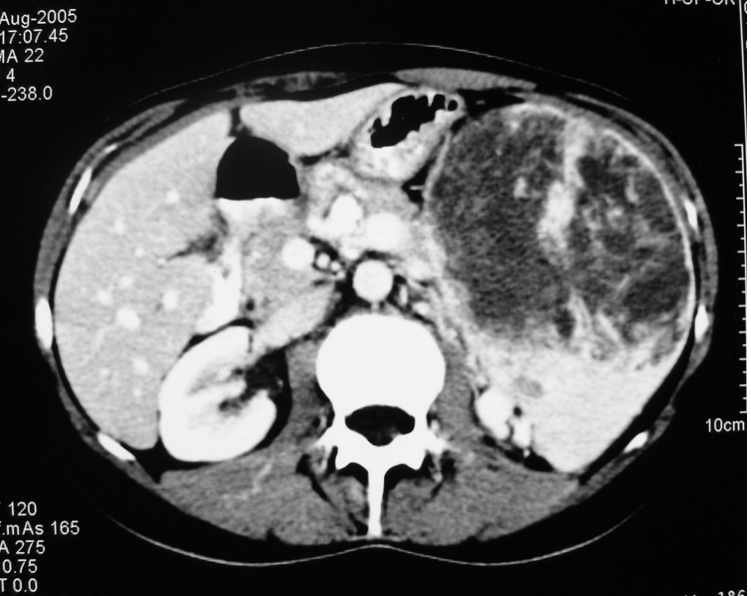
**Computed tomography appearance of giant splenic hemangioma and liver hemangiomatosis**. Cavernous hemangioma of the spleen and multiple hepatic cavernous hemangiomas displayed in a computed tomography hypodense image after IV contrast injection and peripheral, centripetal enhancement.

The CT scan also showed a large hemangioma-like formation in the left abdomen. It was difficult to determine whether this originated from the spleen or from the left adrenal gland, and a large cyst of the right ovary, suspicious of endometriosis (Figure 1), was also observed. The MRI scan, undertaken subsequently, was no more helpful and also raised the suspicion of an adrenal tumor.

**Figure 2:**
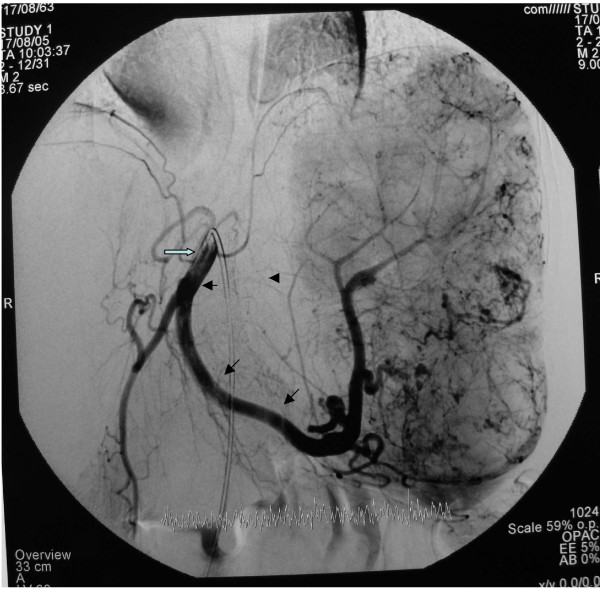
**Angiographic appearance of cavernous splenic hemangioma**. 1. Splenic artery (black arrows). 2. Celiac trunk (white arrow).

Selective arteriography of renal arteries, SMA (superior mesenteric artery), and the celiac trunk, undertaken to clarify the vascularity of the lesion, showed a giant splenic hemangioma with its vascular supply deriving from the splenic artery (Figure 2).

**Figure 3:**
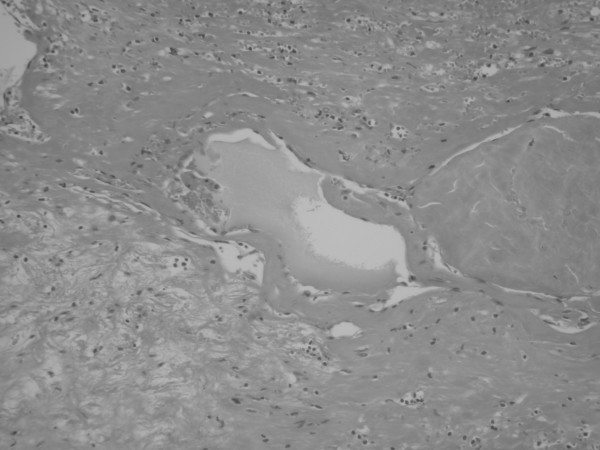
**Pathology highlighted results**. Cavernous splenic hemangioma: multiple blood-filled spaces (lakes), with flat endothelial cells and areas of hematoma.

We decided to proceed to splenectomy because of the possibility of malignant transformation and of rupture and massive bleeding, complications which have been reported by other authors [1,3,4]. The patient underwent elective splenectomy and right ovarectomy. The spleen was 20 cm at its largest dimension, with areas of rupture and creation of local central hematomas (Figure 3). Pre-splenectomy vaccination was administered 2 weeks prior to surgery.

Histology of the spleen showed a large hemangioma with central foci of hematoma (Figure 3). At the periphery of the hematoma and inside the splenic parenchyma, there were many blood-filled spaces surrounded by dilated vessels (Figure 3). The internal surface of the ovarian cyst was lined with cylindrical endometrial epithelium and with endometrial stroma and occasional endometrial glands underneath. The histological findings confirmed the diagnosis of a cavernous hemangioma with hemorrhage and hematoma formation and of an ovarian cyst with endometriosis. The postoperative course was uneventful and 1 year after surgery the patient has remained symptom free.

## Discussion

The first successful surgery for splenic hemangioma was reported by Hodge in 1895. Hemangiomas constitute the majority of benign hepatic neoplasms and are nine times more frequent in females than in males [1]. Arising from sinusoidal epithelium, splenic hemangiomas are thought to be congenital in origin. Most cases are sporadic; however, occasionally they are inherited in autosomal dominant fashion with moderate to high rates of penetrance [5].

Hemangiomas are often asymptomatic and undergo enlargement in fewer than 10% of cases, manifesting as a palpable non-tender mass in the left upper quadrant. Very rarely, splenic hemangiomas may become as large as 25 cm [4]. Generalized splenomegaly may be present, but blood tests are often normal. Splenic hemangioma may occur as part of generalized angiomatosis, as seen in Klippel-Trenaunay syndrome or Kasabach-Meritt syndrome [6,7]. There was no clinical or laboratory evidence associating either syndrome to our case. Nevertheless there are some reports in the literature that describe the synchronous presence of liver and spleen hemangioma [8].

CT and MRI failed to achieve appreciable sensitivity and specificity to define the origin of the giant splenic hemangioma we describe. Velkova and Neveda report a combined sensitivity of 61.3% for ultrasound and CT scan in the recognition of liver and spleen hemangiomas, leaving a substantial number of cases requiring further investigative procedures [9]. They also emphasize the importance of digital angiography in the accurate diagnosis of hemangiomas. Tarazov et al. [8] and Yamamoto et al. [4] highlight the efficacy of angiography and arterial embolism in the diagnosis and treatment of hemangiomas. The MRI appearance of splenic hemangiomas has been described as being similar to that of hepatic hemangiomas. However, larger hemangiomas may have a variable MRI pattern because of complicating features such as hemorrhage, infarction and thrombosis. Differentiation of a splenic hemangioma from malignancy may not be feasible solely on the basis of MRI. The 99T cm-labeled erythrocyte scintigraphy scan is a useful diagnostic modality for large splenic hemangiomas. Recently the employment of single photon emission computed tomography scanning has enhanced the diagnostic sensitivity of scintigraphy to 90% [10]. An alternative preoperative diagnostic intervention is percutaneous needle biopsy under sonographic guidance, which provides scarce material for histological examination and should not be employed because of the high risk of hemorrhage [10].

The presence of central hematomas in the giant hemangioma we describe showed possible rupture. Serious complications of hemangiomas include rupture and malignant transformation. Spontaneous rupture of a giant hepatic hemangioma (diameter >4 cm) with hemoperitoneum occurs very rarely. Willcox et al. report a spontaneous rupture incidence of 25% [1]. Malignant transformation has been reported to occur more frequently with large or multiple hemangiomas and leads most surgeons to favor splenectomy [1,10]. The latter is considered to be a radical treatment for splenic hemangioma.

Splenectomy may be efficacious in the correction of coagulation abnormalities in Kassabach-Meritt syndrome associated with ruptured giant splenic hemangiomas [6,7]. An alternative approach is partial splenectomy, especially in childhood when the preservation of the spleen as an essential part of the developing immune system appears to be of vital importance. The latter approach is also indicated for hemangiomas partially located in the splenic poles [3]. Embolization of hemangiomas, especially hepatic hemangiomas, has been shown to be as effective as surgical removal and appears to be an appealing strategy taking into consideration the reported 2.4% intra-operative mortality rate of hepatic resection. With regard to splenic hemangioma embolization, a successful partial embolization of the lower pole of the spleen has been reported [11]. When radical surgery (splenectomy) may prove risky because of other comorbidity we feel that embolization of giant splenic hemangiomas may prove as effective as in hepatic hemangiomas. It should to be noted however that embolization is a minimally invasive technique which causes tumor infarction and necrosis, but only occasionally reduction in tumor size [7]. Furthermore embolization of giant or multiple splenic hemangiomas may be used preoperatively as it appears to reduce the risk of intra-operative hemorrhage [4,7,11]. In particular, in cases of synchronous presence of liver and spleen hemangiomas, as in the case reported here, the combination of hepatic artery embolization and splenectomy may be the optimal approach. We decided to undertake splenectomy and observation for the small (<4 cm) hepatic hemangiomas as suggested in the literature [1,3]. Caveats of embolization treatment of hemangiomas are massive tumor necrosis with concomitant hepatic failure (in the case of hepatic hemangiomas); postembolization syndrome (abdominal pain, nausea, vomiting, and fever); the possibility of a long-term revascularization (non radical intervention); and, in a number of cases, no regression of tumor size [3,7,8]. Alternative therapies have been tried including corticosteroids, anticoagulants, antiplatelets, antifibrinolytics, interferon, radiation, or antiangiogenic factors, especially in infants, with controversial results [3].

To our knowledge, there is no specific report of giant splenic hemangiomas associated with administration of oral contraceptives. There are numerous reports of intrahepatic and extrahepatic hemangiomas associated with estrogen administration [2]. The exact incidence is believed to be low. It is most common in women in their late twenties who have been on oral contraceptives (OC) for 7 years or more. Experimental studies have revealed that estrogens augment endothelial cell proliferation, migration and organization into capillary-like structures. In vitro they also promote the proliferation of hemangioma vascular endothelial cells and work synergistically with vascular endothelial growth factor [12]. In another study estrogen receptors were reported in hemangioma tissue, an indication that hemangiomas may be target tissues for estrogens [13]. On this pathophysiological basis we may hypothesize a possible causative correlation between OC and splenic hemangiomas along with hepatic hemangiomas. Our patient had been taking oral estrogens for many years, initially as treatment for infertility and subsequently because of an obscure chronic intermittent pelvic pain. Another important observation by Meyniel et al. is that OC administration increases hemangioma volume and favors rupture [2]. There are also numerous reports on the absence of regression of hemangiomas after OC discontinuation, as in our patient [14]. We feel that the synchronous manifestation of endometriosis and giant splenic hemangioma it is quite random, although it should be noted.

## Conclusion

Estrogen administration represents a predisposing factor associated with the appearance of hemangiomas in solid organs of the abdominal cavity. Giant splenic hemangiomas are extremely rare, and require surgical intervention combined with observation or embolization of possibly synchronous multiple small hepatic hemangiomas. Alternative approaches are preoperative embolization and subsequent surgery, which minimizes the risk of intra-operative bleeding, and partial splenectomy or embolization in case of polar localization of small splenic hemangiomas.

## Abbreviations

CT: computed tomography; OC: oral contraceptives.

## Competing interests

The authors declare that they have no competing interests.

## Authors' contributions

All the authors have been involved in literature search, writing and final reviewing of this manuscript. All authors read and approved the final manuscript.

## Consent

Written informed consent was obtained from the patient for publication of this case report and accompanying images. A copy of the written consent is available for review by the Editor-in-Chief of this journal.
